# Variation in corneal biomechanical properties following continuous cross-linking treatment

**DOI:** 10.1186/s40662-026-00482-4

**Published:** 2026-04-01

**Authors:** ChenHao Zhao, XinYu Yao, YeWei Zhao, HongJiang Wu, JiaHui Zong, XuanYa Tong, Daniela Oehring, Lan Yang, XiaoFei Zhou, Ying Li, YanJie Shen, YuFeng Ye, ShiHao Chen, Jia Qu, QinMei Wang, Ahmed Elsheikh, FangJun Bao

**Affiliations:** 1 National Clinical Research Center for Ocular Diseases, Eye Hospital, Wenzhou Medical University, No. 270 Xueyuanxi Road, Wenzhou, 325027 Zhejiang China; 2 Department of Ophthalmology, Shaoxing People’s Hospital, The First Affiliated Hospital of Shaoxing University, No. 568 Zhongxing North Road, Shaoxing, 312000 Zhejiang China; 3 School of Health Professions, University of Plymouth, Plymouth, PL4 8AA UK; 4 State Key Laboratory of Ophthalmology, Optometry and Vision Science, Eye Hospital, Wenzhou Medical University, No. 270 Xueyuan West Road, Wenzhou, 325027 Zhejiang China; 5 National Engineering Research Center of Ophthalmology and Optometry, Eye Hospital, Wenzhou Medical University, No. 270 Xueyuan West Road, Wenzhou, 325027 Zhejiang China; 6 Zhejiang-United Kingdom Joint Laboratory On Ocular Biomechanics, No. 270 Xueyuan West Road, Wenzhou, 325027 Zhejiang China; 7 The Institute of Ocular Biomechanics, Wenzhou Medical University, No. 270 Xueyuan West Road, Wenzhou, 325027 Zhejiang China; 8 OuJiang Laboratory, Eye Hospital, Wenzhou Medical University, Wenzhou, 325000 China; 9 School of Engineering, University of Liverpool, Liverpool, L69 3GH UK; 10 National Institute for Health Research (NIHR) Biomedical Research Centre for Ophthalmology, Moorfields Eye Hospital NHS Foundation Trust and UCL Institute of Ophthalmology, London, UK; 11 School of Medicine, Shaoxing University, Zhejiang Shaoxing, 312000 China

**Keywords:** Keratoconus, Transepithelial, Phototherapeutic keratectomy, Corneal cross-linking, Biomechanics

## Abstract

**Background:**

This retrospective study aimed to evaluate the efficacy of transepithelial accelerated corneal cross-linking (transEpi ACXL) when applied separately or combined with phototherapeutic keratectomy (transPTK ACXL) in keratoconic eyes using biomechanical parameters.

**Methods:**

The study included eyes with progressive keratoconus treated with either continuous transEpi ACXL or transPTK ACXL with 6 months follow-up. The following parameters were assessed preoperatively and 6 months postoperatively: biomechanically corrected intraocular pressure (bIOP), maximum and mean keratometry (K_max_ and K_m_), minimum and central corneal thickness (MCT and CCT), corneal coma, best-corrected visual acuity (BCVA), deformation amplitude ratio at 2 mm nasal/temporal (DAR2), integrated inverse radius (IIR), stiffness parameter at first applanation (SP-A1), highest concavity time (HCT) and two versions of stress–strain index (SSIv1 and SSIv2).

**Results:**

A total of 75 eyes from 72 patients were included (31 in the transEpi ACXL group and 44 in the transPTK ACXL group). The two groups were comparable in age (23.0 ± 5.0 vs. 21.3 ± 5.2 years) and TKC grade distribution (grades 1–4), with no statistically significant between-group differences (*P* = 0.321 and 0.766, respectively). Both groups exhibited significant reductions in IIR, and increases in SSIv1 and SSIv2 (all *P* < 0.05). The improvements in K_max_, coma, and SSIv2 were greater in the transPTK ACXL group than in the transEpi ACXL group (all *P* < 0.05). The relative difference in SSIv2 (SSIv2_Rdif_) exceeded those of other biomechanical parameters in both groups (transEpi ACXL: SSIv2_Rdif_ > DAR2_Rdif_, IIR_dif_; transPTK ACXL: SSIv2_Rdif_ > DAR2_Rdif_, IIR_Rdif_, SP-A1_Rdif_, HCT_Rdif_, and SSIv1_Rdif_, all *P* < 0.05).

**Conclusions:**

TransPTK ACXL resulted in better biomechanical and visual benefits in comparison with transEpi ACXL 6 months after treatment. SSIv2 demonstrated superior performance in assessing the efficacy of corneal cross-linking.

## Background

Keratoconus is a progressive, typically asymmetric corneal dystrophy characterized by stromal thinning and ectatic protrusion, resulting in irregular astigmatism and substantial vision loss [1]. In the early stages, visual deficits are frequently corrected using spectacles or rigid contact lenses. However, advanced disease may necessitate corneal transplantation to restore functional vision [2]. Corneal cross-linking (CXL) has emerged as the gold-standard intervention for delaying and potentially halting keratoconus progression [3, 4] thereby reducing reliance on transplantation.

Conventional CXL has demonstrated long-term efficacy [5–7], while its accelerated form accelerated corneal cross-linking (ACXL) has addressed the protocol's lengthy surgical duration and low patient comfort [8–11]. Recent advancements have also enabled CXL to be combined with visual error correction surgeries, such as transepithelial phototherapeutic keratectomy (transPTK). This combined approach, known as the Cretan protocol, has shown improvements in both best-corrected visual acuity (BCVA) and keratometric asymmetry, surpassing preoperative values [12–14]. The Cretan protocol involves a 50-µm phototherapeutic keratectomy (PTK), followed by conventional CXL at 3 mW/cm^2^ for 30 min, delivering a total energy of 5.4 J/cm^2^ [15, 16]. Given evidence that higher irradiance can reduce biomechanical efficacy under constant total energy, we used a moderately increased total fluence (7.2 J/cm^2^) to support cross-linking efficiency [17]. We applied a customized PTK ablation depth equal to the central epithelial thickness plus 10 µm within a 9-mm ablation zone, followed by an ACXL protocol, referred to as transPTK ACXL. However, a critical concern is the loss of tissue during the PTK procedure, which inevitably compromises corneal biomechanics and may diminish the mechanical benefits of CXL. Therefore, quantitative analyses of the impact on corneal biomechanics postoperatively are essential for fully understanding these effects.

While combined transPTK ACXL surgeries have demonstrated efficacy in halting keratoconus progression through topographic stabilization [13, 14], key clinical questions remain on how its outcomes compare with those of transepithelial accelerated corneal cross-linking (transEpi ACXL), where only ACXL is applied. This study attempts to address this important question, and in doing so, it seeks to modify the Cretan protocol to guide the implementation of the combined protocol in keratoconic cases.

## Methods

### Patient inclusion and exclusion

This retrospective study received approval from the Ethics Committee of the Eye Hospital of Wenzhou Medical University (H2023-017-K-14) and was conducted in accordance with the Declaration of Helsinki. Informed consent was obtained from all participants for the use of their clinical data in research. We retrospectively analyzed the medical records of 72 patients (75 eyes) diagnosed with progressive keratoconus, comprising two treatment cohorts: 31 patients (31 eyes) treated with transEpi ACXL and 41 patients (44 eyes) treated with transPTK ACXL. Surgical technique selection was guided by preoperative corneal morphology, systemic health status, and surgeon assessment of individualized therapeutic requirements. Specifically, eyes with central residual stromal thickness (central corneal thickness [CCT] − central epithelial thickness − 10 µm) < 400 µm were advised to undergo transEpi ACXL to minimize the risk of stromal damage. Eyes with thicker corneas that tolerated epithelial removal and preferred for surface smoothing were recommended for transPTK ACXL. Patients were informed of the potential benefits and risks of both procedures and made the final decision. Detailed procedural parameters are summarized in Table 1.

**Table 1:** Corneal cross-linking methodology

Parameter	transEpi ACXL	transPTK ACXL
Treatment target	Progressive keratoconus	Progressive keratoconus
Fluence (J/cm^2^)	7.2	7.2
Soak time and interval time (min)	30 min, q1	30 min, q1
Intensity (mW)	10	10
Treatment time (min)	12	12
Epithelium status	Epithelium-on	PTK mode (Schwind Amaris 1050RS, Germany)
Chromophore	Riboflavin (Peschke, Switzerland)	Riboflavin (Peschke, Switzerland)
Chromophore carrier	HPMC	HPMC
Chromophore osmolarity	iso-osmolar	iso-osmolar
Chromophore concentration	0.25%	0.1%
Light source (device name)	Avedro (Avedro KXL System)	Avedro (Avedro KXL System)
Irradiation mode (interval)	Continuous	Continuous

*transEpi ACXL* = transepithelial accelerated corneal cross-linking; *transPTK ACXL* = transepithelial phototherapeutic keratectomy combined with accelerated corneal cross-linking; *q1* = quaque 1 min

Inclusion in the study required documented disease progression over a 12-month period, defined by one or more of the following: (1) an increase of ≥ 1.00 D in maximum keratometry (K_max_), (2) an increase of ≥ 1.00 D in manifest astigmatism, or (3) a decrease of ≥ 10 µm in minimum corneal thickness (MCT) [6, 18, 19]. The exclusion criteria included: (1) CCT < 400 µm, (2) endothelial cell density < 2000 cells/mm^2^, (3) significant corneal scarring or active ocular surface disease, (4) systemic autoimmune disorders, and (5) pregnancy or lactation.

### TransEpi ACXL protocol

Following topical anesthesia with 0.01% proparacaine hydrochloride (Alcaine, Alcon, USA), an eyelid speculum was inserted. Transepithelial riboflavin was delivered as 0.25% riboflavin (Peschke, Switzerland) to facilitate epithelial penetration. After confirming complete stromal saturation, the corneal surface was thoroughly rinsed with saline. Ultraviolet-A (UVA) irradiation (365 nm wavelength) was delivered using the Avedro KXL system (Avedro Inc., USA) at a continuous irradiance of 10 mW/cm^2^ with a 9-mm diameter beam, delivering a total fluence of 7.2 J/cm^2^. Postoperatively, a bandage contact lens (Bausch & Lomb, USA) was inserted and retained for 1–3 days. 0.1% fluorometholone eye drops were instilled 4 times a day for 1 week, followed by a weekly tapering regimen over 4 weeks. Tobramycin and artificial tears were administered 4 times daily for 1 month.

### TransPTK ACXL protocol

Several modifications were made to the transPTK ACXL protocol based on the Cretan protocol [15]. Transepithelial PTK was performed on the Schwind Amaris 1050RS excimer laser (Schwind Eye-Tech-Solutions, Germany) in “transPTK smoothing” mode with a 9-mm ablation zone centered on the corneal apex. The programmed ablation depth was defined as the OCT-measured central epithelial thickness plus 10 µm and was applied uniformly across the ablation zone (Optovue Solix, Optovue Inc., USA). Stromal saturation was subsequently achieved through 30-min application of 0.1% riboflavin (Peschke, Switzerland). Eligibility required a projected central residual stromal thickness of more than 400 µm after simulated removal of the epithelium plus 10 µm. The ACXL step used an Avedro KXL system (Avedro Inc., USA) at 365 nm UVA wave length, continuous 10 mW/cm^2^ energy for 12 min (7.2 J/cm^2^). In this system, the beam profile was verified for homogeneity, and in-procedure irradiance calibration. A bandage contact lens (Bausch & Lomb, USA) was applied until complete re-epithelialization (3–7 days). Postoperative 0.1% fluorometholone eye drops were applied four times daily for one month and tapered by one application per month thereafter. Artificial tears and tobramycin regimens were identical to those used in the transEpi ACXL group.

### Data collection

Demographic and clinical baseline data were systematically documented, including sex, age, medical comorbidities, and lifestyle factors. BCVA and slit-lamp biomicroscopy were implemented. Corneal biomechanical profiling was conducted using the Corneal Visualization Scheimpflug Technology (Corvis ST; Oculus Optikgeräte GmbH, Germany), with the following parameters quantified: biomechanically corrected intraocular pressure (bIOP), apex and deformation amplitude ratio at 2 mm nasal/temporal (DAR2), integrated inverse radius (IIR), stiffness parameter at first applanation (SP-A1), highest concavity time (HCT) and stress–strain index (SSIv1 and SSIv2). Only acquisitions with “OK” quality and no dropouts were analyzed.

Topometric characterization was conducted using Pentacam Scheimpflug imaging (Oculus Optikgeräte GmbH, Germany), capturing K_max_, mean keratometry (K_m_), MCT, CCT, and topographical keratoconus classification (TKC) grade. Keratoconus pattern was classified as central or paracentral according to the location of the cone apex, defined as the point of the maximum corneal elevation [20]. Zernike coefficients from third- to eighth-order aberrations were used to systematically quantify coma in the central 6 mm of the cornea. The root mean square (RMS) values of four parameters—third-order vertical coma (Z_3_^−1^), third-order horizontal coma (Z_3_^1^), fifth-order vertical coma (Z_5_^−1^), and fifth-order horizontal coma (Z_5_^1^)—were calculated as key indicators for characterizing anterior corneal surface coma. Measurements were obtained at the preoperative baseline and at the 6-month postoperative follow-up and the changes between these two time points were calculated. Only scans with a quality score (QS) marked as "OK" were included in the analysis.

### *Relative difference of biomechanical parameters (X*_*Rdif*_*)*

Normalized relative difference were computed to enable cross-scale comparisons of biomechanical alterations following treatment. For each biomechanical parameter (DAR2, IIR, SP-A1, HCT, SSIv1 and SSIv2), the relative difference (X_Rdif_) was defined as:

$${X}_{Rdif} = \frac{{X}_{dif}}{{X}_{pre}} \times 100\%$$

where:

X_pre_ denotes the preoperative baseline, and X_dif_ represents the difference between 6-month postoperative and preoperative measurements. Parameter-specific derivatives were denoted using subscript notation (e.g., SSIv2_Rdif_, SP-A1_Rdif_). Positive values of SSIv2_Rdif_, SSIv1_Rdif_, SP-A1_Rdif_ indicate increased corneal stiffness; negative values of DAR2_Rdif_, IIR_Rdif_, HCT_Rdif_ are consistent with stiffening.

### Statistical analyses

Statistical analyses were performed using IBM SPSS Statistics version 26.0 (IBM Corp., USA). Continuous variables were reported as mean ± standard deviation, and categorical variables as frequencies. Baseline characteristics were compared using independent Student’s *t*-tests and Pearson’s Chi-squared tests. Longitudinal changes in biomechanical parameters from preoperative to 6-month postoperative intervals were analyzed using generalized estimating equation (GEE). Intergroup differences in dynamic corneal response (DCR) parameters were analyzed using propensity score matching (PSM). After PSM, independent-sample *t*-tests were performed for normally distributed variables, and Mann–Whitney U tests were applied for non-normally distributed variables. For SSIv1 and SSIv2, intergroup differences were assessed using analysis of covariance (ANCOVA). Treatment group was included as the fixed factor, and baseline TKC grade was included as covariates. Nonparametric comparisons of normalized relative differences (X_Rdif_) across biomechanical indices were performed using Friedman tests with post hoc Dunn-Bonferroni corrections. Sample size estimation was performed using G*Power software (version 3.1; Franz Faul, University of Kiel) with an alpha level of 0.05 and a statistical power of 90%. Statistical significance was defined as two-tailed *P* < 0.05.

## Results

### Demographic and clinical characteristics

This retrospective study enrolled 72 consecutive keratoconus patients (75 eyes) undergoing two ACXL protocols: transEpi ACXL (31 eyes) and transPTK ACXL (44 eyes). All patients in both treatment groups were classified as having central keratoconus. Baseline demographic and clinical characteristics demonstrated comparable distributions between cohorts in age (*P* = 0.321), bIOP (*P* = 0.118), K_max_ (*P* = 0.089), K_m_ (*P* = 0.051), corneal coma (*P* = 0.799), BCVA (*P* = 0.419) and TKC grade (*P* = 0.766). However, significant differences in baseline MCT (*P* = 0.006) and CCT (*P* = 0.003) were observed between the groups (Table 2).

**Table 2:** Patient demographic information and clinical characteristics

Parameter	transEpi ACXL	transPTK ACXL	*P* value
No. of eyes	31	44	–
Age (years)	23.0 ± 5.0	21.3 ± 5.2	0.321
bIOP (mmHg)	13.0 ± 2.2	14.0 ± 2.4	0.118
K_max_ (D)	60.6 ± 9.9	56.6 ± 6.2	0.089
K_m_ (D)	50.0 ± 5.7	47.4 ± 3.2	0.051
MCT (μm)	446.1 ± 39.9	470.8 ± 32.9	**0.006**
CCT (μm)	468.0 ± 38.1	492.4 ± 25.9	**0.003**
Coma (μm)	2.7 ± 1.1	2.8 ± 1.5	0.799
BCVA (logMAR)	0.30 (0.10, 0.40)	0.22 (0.10, 0.30)	0.419
TKC grade (1/2/3/4)	3/7/11/10	6/11/19/8	0.766

*transEpi ACXL* = transepithelial accelerated corneal cross-linking; *transPTK ACXL* = transepithelial phototherapeutic keratectomy combined with accelerated corneal cross-linking; *bIOP* = biomechanically corrected intraocular pressure; *K*_*max*_ = maximum keratometry; *K*_*m*_ = mean keratometry; *MCT* = minimum corneal thickness; *CCT* = central corneal thickness; *BCVA* = best-corrected visual acuity; *TKC* = topographical keratoconus classification

*P* values in bold indicate statistical significance

In the transPTK ACXL group, mean K_max_, CCT, MCT and corneal coma significantly decreased at 6 months postoperatively, while bIOP increased (all *P* < 0.05). In the transEpi ACXL group, CCT also showed a significant decrease, and BCVA improved at the 6-month follow-up (both *P* < 0.05). Changes in bIOP (*P* = 0.503), K_m_ (*P* = 0.157), CCT (*P* = 0.512), BCVA (*P* = 0.444), spherical refraction (*P* = 0.682) and cylindrical refraction (*P* = 0.710) from baseline to 6 months postoperatively did not differ significantly between the transEpi ACXL and transPTK ACXL groups. In contrast, the reduction in K_max_ (*P* = 0.011), MCT (*P* = 0.006) and corneal coma (*P* = 0.016) was significantly greater in the transPTK ACXL group than in the transEpi ACXL group (Table 3).

**Table 3:** Comparison of the difference in topographic, visual and biomechanical outcomes between preoperative and 6-month postoperative measurements across groups

Parameter	transEpi ACXL	transPTK ACXL	*P* value
bIOP_dif_ (mmHg)	0.60 ± 1.60	0.84 ± 2.19	0.503
K_maxdif_ (D)	0.57 ± 3.60	− 1.78 ± 3.52	**0.011**
K_mdif_ (D)	0.27 ± 1.76	− 0.51 ± 2.31	0.157
MCT_dif_ (μm)	− 5.23 ± 17.03	− 17.22 ± 16.14	**0.006**
CCT_dif_ (μm)	− 7.53 ± 17.69	− 10.63 ± 18.23	0.512
Coma_dif_ (μm)	− 0.05 ± 0.24	− 0.34 ± 0.55	**0.016**
BCVA_dif_ (logMAR)	0.00 (− 0.13, 0.00)	0.00 (− 0.12, 0.05)	0.444
Sphere_dif_ (D)	0.40 ± 1.18	0.57 ± 1.80	0.682
Cylinder_dif_ (D)	0.15 ± 1.18	0.28 ± 1.70	0.710
DAR2_dif_^a^	− 0.08 (− 0.96, 0.21)	− 0.16 (− 0.36, 0.51)	0.318
IIR_dif_ (mm^−1^)^a^	− 0.85 ± 1.27	− 0.67 ± 1.22	0.639
SP-A1_dif_ (mmHg/mm)^a^	− 0.74 ± 8.00	3.88 ± 16.40	0.308
HCT_dif_ (ms)^a^	0.33 (− 0.61, 0.59)	− 0.14 (− 0.81, 0.54)	0.650
SSIv1_dif_	0.06 ± 0.12	0.08 ± 0.12	0.969
SSIv2_dif_	0.05 ± 0.06	0.12 ± 0.10	**0.017**

*transEpi ACXL* = transepithelial accelerated corneal cross-linking; *transPTK ACXL* = transepithelial phototherapeutic keratectomy combined with accelerated corneal cross-linking; *bIOP* = biomechanically corrected intraocular pressure; *K*_*max*_ = maximum keratometry; *K*_*m*_ = mean keratometry; *MCT* = minimum corneal thickness; *CCT* = central corneal thickness; *BCVA* = best-corrected visual acuity; *DAR2* = deformation amplitude ratio at 2 mm nasal/temporal; *IIR* = integrated inverse radius; *SP-A1* = stiffness parameter at first applanation; *HCT* = highest concavity time; *SSIv1* = the earlier stress–strain index; *SSIv2* = the updated stress–strain index; *X*_*dif*_ = the changes in topographic, visual and biomechanical parameters between 6-month postoperative and preoperative measurements

*P* values in bold indicate statistical significance

^a^Data after propensity score matching (PSM)

### Changes in biomechanical parameters between baseline and 6 months post-surgery

Adjusted for CCT_dif_ and bIOP_dif_, no significant temporal differences in DAR2, SP-A1 or HCT were identified between preoperative and 6-month postoperative assessments within either treatment cohort (all *P* > 0.05; Table 4). In contrast, IIR decreased significantly post-intervention in both groups after adjustments for CCT_dif_ and bIOP_dif_ (both *P* < 0.001; Table 4). Stress–strain indices (SSIv1 and SSIv2) showed progressive increases from preoperative to 6-month postoperative assessments for both CXL protocols, after adjusting for CCT_dif_ and bIOP_dif_ (all *P* < 0.01; Table 4).

**Table 4:** Biomechanical parameters before and 6 months after ACXL of two protocols

Parameter	transEpi ACXL			transPTK ACXL		
	Pre	Pos6m	*P* value	Pre	Pos6m	*P* value
DAR2	6.48 ± 1.43	6.28 ± 1.29	0.062	5.92 ± 1.15	5.76 ± 1.18	0.548
IIR	13.47 ± 2.36	12.73 ± 2.03	** < 0.001**	12.51 ± 1.91	11.49 ± 1.95	** < 0.001**
SP-A1	50.08 ± 24.57	48.94 ± 22.53	0.807	61.35 ± 20.02	64.09 ± 17.68	0.881
HCT	18.38 ± 0.82	18.28 ± 0.80	0.816	18.44 ± 0.82	18.34 ± 0.73	0.530
SSIv1	0.70 ± 0.10	0.75 ± 0.15	** < 0.001**	0.73 ± 0.12	0.79 ± 0.15	**0.004**
SSIv2	0.57 ± 0.12	0.62 ± 0.13	**0.001**	0.56 ± 0.12	0.67 ± 0.13	** < 0.001**

*DAR2* = deformation amplitude ratio at 2 mm nasal/temporal; *IIR* = integrated inverse radius; *SP-A1* =stiffness parameter at first applanation; *HCT* = highest concavity time; *SSIv1* = the earlier stress–strain index; *SSIv2* = the updated stress–strain index; *transEpi ACXL* = transepithelial accelerated corneal cross-linking; *transPTK ACXL* = transepithelial phototherapeutic keratectomy combined with accelerated corneal cross-linking; *Pre* = preoperative; *Pos6m* = 6-month postoperative

*P* value: comparison of the preoperative vs. 6-month postoperative values in the same group

### Comparative biomechanical outcomes between transEpi ACXL and transPTK ACXL cohorts

After PSM, no significant differences were observed between the groups in DAR2, IIR, SP-A1, or HCT (all *P* > 0.05, Table 3). The transPTK ACXL cohort demonstrated a significantly greater SSIv2 elevation compared to the transEpi ACXL group after adjustment for preoperative TKC grade (*P* = 0.017, Table 3). No statistically significant intergroup difference was observed in SSIv1 following TKC adjustment (*P* =0.969, Table 3).

### Normalized relative differences across six biomechanical indices

Normalized relative differences (X_Rdif_) were calculated to standardize postoperative changes in biomechanical parameters (Table 5). In the transEpi ACXL group, SSIv2_Rdif_ exhibited greater changes than IIR_Rdif_ and DAR2_Rdif_; while SSIv1_Rdif_ showed greater changes than IIR_Rdif_ (all *P* < 0.05; Table 5). In the transPTK ACXL group, stress-strain indices (both SSIv2_Rdif_ and SSIv1_Rdif_) were significantly higher than those of the other parameters (SSIv2_Rdif_ > DAR2_Rdif_, IIR_Rdif_, SP-A1_Rdif_, HCT_Rdif_ and SSIv1_Rdif_; SSIv1_Rdif_ > IIR_Rdif_; all *P*<0.05; Table 5).

**Table 5:** Normalized relative differences (X_Rdif_) across 6 biomechanical indices

Parameter	transEpi ACXL	transPTK ACXL
DAR2	− 0.01 (− 0.13, 0.06)	− 0.02 (− 0.07, 0.09)
IIR	− 0.06 (− 0.10, 0.02)	− 0.06 (− 0.16, − 0.02)
SP-A1	0.02 (− 0.12, 0.12)	0.08 (− 0.13, 0.24)
HCT	0.01 (− 0.04, 0.03)	− 0.01 (− 0.05, 0.03)
SSIv1	0.03 (− 0.03, 0.23)	0.07 (0.003, 0.17)
SSIv2	0.12 (0.06, 0.15)	0.14 (0.06, 0.33)
*P* value	** < 0.001**	** < 0.001**

*DAR2* = deformation amplitude ratio at 2 mm nasal/temporal; *IIR* = integrated inverse radius; *SP-A1* = stiffness parameter at first applanation; *HCT* = highest concavity time; *SSIv1* = the earlier stress–strain index; *SSIv2* = the updated stress–strain index; *transEpi* *ACXL* = transepithelial accelerated corneal cross-linking; *transPTK ACXL* = transepithelial phototherapeutic keratectomy combined with accelerated corneal cross-linking; *X*_*Rdif*_ = the normalized relative difference of six biomechanical parameters between 6-month postoperative and preoperative measurements

*P* value: comparison of the normalized relative difference in six biomechanical parameters (X_Rdif_)

## Discussion

This study aimed to evaluate the biomechanical effects of two CXL techniques—transEpi ACXL and transPTK ACXL—in progressive keratoconus eyes. The results indicated that both techniques significantly improved corneal biomechanics, but transPTK ACXL provided superior outcomes, particularly in increasing corneal stiffness. This discussion focuses on the comparison between these two approaches, examining their biomechanical outcomes, tissue loss concerns, visual improvement and overall efficacy in managing keratoconus progression.

Both transEpi ACXL and transPTK ACXL resulted in notable improvements in corneal biomechanical parameters, such as the IIR and stress–strain index (SSIv1 and SSIv2). However, transPTK ACXL demonstrated more substantial gains in certain parameters, most notably in SSIv2, which serves as a more refined measure of corneal stiffness. The SSIv2 in the transPTK ACXL group increased significantly from 0.56 to 0.67, compared with the increase in the transEpi ACXL group from 0.57 to 0.62. This difference suggests that transPTK ACXL leads to a more substantial improvement in corneal stiffness, which is critical for halting the progression of keratoconus and preventing the need for corneal transplantation.

SSIv1 was originally developed to estimate corneal stiffness in healthy eyes [21]. In contrast, SSIv2 incorporates the biomechanical characteristics of abnormal corneas [22]. Therefore, SSIv2 has been identified as a highly sensitive index for evaluating corneal biomechanical properties [21, 22]. Its superior sensitivity to cross-linking-induced changes makes it an ideal metric for assessing the effectiveness of CXL techniques. The observed greater improvement in the SSIv2 in the transPTK ACXL cohort could be attributed to the additional epithelial removal step in this technique, which may enhance the penetration of riboflavin into the corneal stroma, and thus improve the efficacy of cross-linking [15, 23].

On the other hand, transEpi ACXL, which preserves the epithelium, also resulted in favorable changes in biomechanical parameters, but the improvements were less pronounced compared with transPTK ACXL. This suggests that while transEpi ACXL is effective, it may not achieve the same level of biomechanical enhancement as transPTK ACXL due to the limited penetration of riboflavin in the absence of epithelial removal [15, 24, 25]. The epithelium acts as a diffusion barrier for riboflavin and reduces effective stromal irradiance. Moreover, the Bunsen-Roscoe reciprocity law is limited by oxygen depletion at higher irradiances. Pulsed delivery and oxygen supplementation can partially restore oxygen and improve cross-linking efficiency [26, 27]. In addition, baseline pachymetric differences between groups should be considered when interpreting biomechanical outcomes. The transEpi ACXL group had thinner corneas preoperatively, which may have contributed to the fewer biomechanical changes observed after surgery. Although ANCOVA adjustment was performed, residual effects might not be fully excluded.

One of the main concerns with transPTK ACXL is the tissue loss associated with the PTK step. Here, the transPTK ACXL group exhibited a greater loss of MCT compared with transEpi ACXL group, raising concerns about long-term corneal stability [28, 29]. The ablation depth, defined as the central epithelial thickness plus 10 µm, was centered on the corneal apex. Localized epithelial thinning at the MCT and K_max_ region is common in keratoconus [30, 31]. Therefore, PTK may remove more stromal tissue than intended over the apex. This mechanism could partly explain the greater MCT reduction observed in this group. Even so, the average MCT thinning in the transPTK ACXL group was 17.22 µm, which remained within a clinically acceptable range. Moreover, transPTK ACXL still offer advantages in improving corneal topography, as it demonstrated greater flattening in K_max_ [12, 13, 28].

In contrast, transEpi ACXL avoids epithelial removal, preserves corneal tissue and reduces the risk of inducing additional corneal thinning. This makes transEpi ACXL an attractive option for patients with thinner corneas, as it mitigates the risk of compromising the cornea’s structural integrity. However, the trade-off is the slightly less effective riboflavin penetration and the subsequent decrease in the overall biomechanical benefit of the procedure [32].

In addition, previous studies have suggested that transPTK ACXL leads to more significant improvements in visual quality parameters [28]. This may be attributed to the smoother and more regular corneal surface created by the PTK procedure, which reduces corneal irregularities and enhances visual quality. Although the present study did not show a significant difference in BCVA, spherical and cylindrical refraction between the two techniques, transPTK ACXL demonstrated greater reduction in K_max_ and corneal coma, suggesting superior postoperative visual quality. This may be explained by the limited depth of stromal ablation (10 µm beyond epithelial removal), which is unlikely to induce meaningful refractive changes. In addition, potential improvements in optical quality, such as reduced higher-order aberrations, may not be fully captured by standard BCVA or refraction measurements.

In general, both transEpi ACXL and transPTK ACXL have demonstrated effectiveness in halting the progression of keratoconus. The additional PTK step in transPTK ACXL may contribute to better visual outcomes, as it helps to address both the ectatic corneal surface and the biomechanical weaknesses characteristic of keratoconus. The combined approach not only stabilizes the cornea but also improves corneal optical irregularities, as supported by prior studies [28]. The reduction in aberrations, combined with biomechanical stabilization, may ultimately result in better long-term visual outcomes for patients undergoing transPTK ACXL. Our use of 7.2 J/cm^2^ at 10 mW/cm^2^ for transEpi ACXL and transPTK ACXL is supported by contemporary studies using comparable protocols with similar irradiance levels and total fluence, which have demonstrated effective keratoconus stabilization with acceptable safety profiles [11, 33, 34].

The safety profile of both techniques was generally favorable, with minimal complications. In terms of postoperative recovery, the transEpi ACXL group may have an edge in terms of comfort. Preservation of the epithelium leads to faster re-epithelialization and less postoperative pain compared to transPTK ACXL, which involves a longer recovery time due to epithelial healing [35, 36]. However, this advantage may come at the cost of a slightly less effective treatment outcome in terms of biomechanical stabilization. Although we did not use mitomycin C (MMC), we recognize its prophylactic role after surface ablation at greater stromal exposure depths. We now specify criteria under which MMC 0.02% (10–20 s) would be considered (e.g., anticipated ablation encroaching on Bowman’s at the cone apex or patient-level haze risk) [37].

Both techniques require the use of bandage contact lenses and a strict postoperative medication regimen, including antibiotics and steroids, to manage inflammation and prevent infection. Postoperative haze was graded on the Fantes scale at each visit [38]. Mild adverse effects, manifested as transient haze of grade ≤ 1, occurred in 14 eyes (45.2%) in the transEpi ACXL group and 30 eyes (68.2%) in the transPTK ACXL group at 1 month post-surgery. All cases resolved by 6 months. No significant complications, including visually significant haze or scarring, were observed. Endothelial cell density did not change significantly between baseline and 6 months in either group. The relatively short recovery period (1–3 days for transEpi ACXL and 3–7 days for transPTK ACXL) aligns with the mild adverse event profiles reported in this study, with no patients experiencing significant complications.

While this study provides valuable insights into the comparative efficacy of transEpi ACXL and transPTK ACXL, several limitations must be considered. The relatively short 6-month follow-up period may not capture long-term changes in corneal biomechanics or the durability of the improvements in stiffness. A longer follow-up period is necessary to fully understand the long-term stability of both procedures. This long-term study is underway. Moreover, the small sample size within each TKC stage limits the statistical power for stage-stratified analysis of treatment efficacy. Larger, stage-matched studies are required to clarify the stage-specific effects of CXL. Furthermore, the retrospective design of this study may introduce potential selection bias in treatment allocation. Future prospective randomized studies with larger sample sizes and more diverse populations are needed to confirm these results and explore the long-term outcomes of both techniques.

## Conclusions

Both transPTK ACXL and transEpi ACXL are effective in halting the progression of keratoconus and improving corneal biomechanical properties. However, transPTK ACXL appears to offer superior biomechanical improvements, particularly in terms of corneal stiffness. While the PTK component in transPTK ACXL raises concerns about tissue loss, this technique may be more beneficial for patients seeking better visual outcomes. TransEpi ACXL, on the other hand, provides a safer, less invasive alternative, particularly for patients with thinner corneas. Among the parameters evaluated, SSIv2 demonstrated a strong sensitivity to reflect the biomechanical outcomes of CXL. Ultimately, the choice between these two techniques should be guided by the individual patient’s corneal morphology, disease severity, and long-term goals for visual and biomechanical outcomes. In summary, transPTK ACXL may be considered for selected patients to enhance postoperative surface regularization and biomechanical stability, whereas transEpi ACXL remains a safer and less invasive option for patients with thinner corneas.

## Data Availability

The datasets used and/or analyzed during the current study are available from the corresponding author on reasonable request.

## Abbreviations

ACXL: Accelerated corneal cross-linking
BCVA: Best-corrected visual acuity
bIOP: Biomechanically corrected intraocular pressure
CCT: Central corneal thickness
CXL: Corneal cross-linking
DAR2: Deformation amplitude ratio at 2 mm nasal/temporal
HCT: Highest concavity time
IIR: Integrated inverse radius
K_m_: Mean keratometry
K_max_: Maximum keratometry
MCT: Minimum corneal thickness
MMC: Mitomycin C
SP-A1: Stiffness parameter at first applanation
SSIv1: The earlier stress–strain index
SSIv2: The updated stress–strain index
TKC: Topographical keratoconus classification
transEpi ACXL: Transepithelial accelerated corneal cross-linking
transPTK: Transepithelial phototherapeutic keratectomy
X_dif_: Difference between 6-month postoperative and preoperative measurement of parameters
X_pre_: Preoperative baseline of parameters
X_Rdif_: Relative difference of parameters
